# Public Awareness of Melioidosis in Thailand and Potential Use of Video Clips as Educational Tools

**DOI:** 10.1371/journal.pone.0121311

**Published:** 2015-03-24

**Authors:** Praveen Chansrichavala, Nittayasee Wongsuwan, Suthee Suddee, Mayura Malasit, Maliwan Hongsuwan, Prapass Wannapinij, Rungreung Kitphati, Nicholas P. J. Day, Susan Michie, Sharon J. Peacock, Direk Limmathurotsakul

**Affiliations:** 1 Mahidol-Oxford Tropical Medicine Research Unit, Faculty of Tropical Medicine, Mahidol University, Bangkok, 10400, Thailand; 2 Warinchamrab Hospital, Ubon Ratchathani, 34190, Thailand; 3 Bureau of Emerging Infectious Disease, Department of Disease Control, Ministry of Public Health, NonThaburi, 11000, Thailand; 4 Centre for Tropical Medicine, Nuffield Department of Medicine, University of Oxford, Old Road Campus, Roosevelt Drive, Oxford, OX3 7FZ, United Kingdom; 5 Centre for Outcomes Research Effectiveness, Research Department of Clinical, Educational and Health Psychology, University College London, London, WC1E 6BT, United Kingdom; 6 Department of Medicine, University of Cambridge, Addenbrooke’s Hospital, Cambridge, CB2 OQQ, United Kingdom; 7 Department of Tropical Hygiene, Faculty of Tropical Medicine, Mahidol University, Bangkok, 10400, Thailand; University of California San Diego, UNITED STATES

## Abstract

**Background:**

Melioidosis causes more than 1,000 deaths in Thailand each year. Infection occurs via inoculation, ingestion or inhalation of the causative organism (*Burkholderia pseuodmallei*) present in soil and water. Here, we evaluated public awareness of melioidosis using a combination of population-based questionnaire, a public engagement campaign to obtain video clips made by the public, and viewpoints on these video clips as potential educational tools about the disease and its prevention.

**Methods:**

A questionnaire was developed to evaluate public awareness of melioidosis, and knowledge about its prevention. From 1 March to 31 April 2012, the questionnaire was delivered to five randomly selected adults in each of 928 districts in Thailand. A video clip contest entitled “Melioidosis, an infectious disease that Thais must know” was run between May and October 2012. The best 12 video clips judged by a contest committee were shown to 71 people at risk from melioidosis (diabetics). Focus group interviews were used to evaluate their perceptions of the video clips.

**Results:**

Of 4,203 Thais who completed our study questionnaire, 74% had never heard of melioidosis, and 19% had heard of the disease but had no further knowledge. Most participants in all focus group sessions felt that video clips were beneficial and could positively influence them to increase adherence to recommended preventive behaviours, including drinking boiled water and wearing protective gear if in contact with soil or environmental water. Participants suggested that video clips should be presented in the local dialect with simple words rather than medical terms, in a serious manner, with a doctor as the one presenting the facts, and having detailed pictures of each recommended prevention method.

**Conclusions:**

In summary, public awareness of melioidosis in Thailand is very low, and video clips could serve as a useful medium to educate people and promote disease prevention.

**Presented in Part:**

World Melioidosis Congress 2013, Bangkok, Thailand, 18–20 September 2013 (abstract OS VII-04).

## Introduction

Melioidosis is a serious community-acquired infectious disease caused by the Gram-negative bacillus *Burkholderia pseudomallei*, which is present in soil and water in a defined geographic distribution [1, 2]. Clinical presentation may range from acute, fulminant septicaemia to chronic cough with pulmonary infiltration similar to tuberculosis. In northeast Thailand, melioidosis is the second most common cause of community-acquired bacteremia with an overall case fatality rate of 43% [3, 4]. It is estimated that melioidosis causes more than 1,000 deaths per year in northeast Thailand, and the number of people dying there from melioidosis is now comparable to deaths from tuberculosis, and exceeds those from malaria, diarrheal illnesses and measles combined [4]. Diabetes mellitus is the major underlying risk factor for melioidosis, and is present in more than 50% of all melioidosis cases [5]. The risk of diabetics acquiring melioidosis is about 12 times higher compared with non-diabetics [4, 6], and diabetics represent the major target population for melioidosis preventive measures [7].

Melioidosis should be preventable since infection occurs as a direct result of exposure to *B*. *pseudomallei* in the environment. Infection is acquired through bacterial inoculation, contamination of wounds, inhalation and ingestion [1]. In Thailand, activities associated with increased risk of melioidosis include working in a rice field and other activities associated with exposure to soil or water, having an open wound, eating food contaminated with soil or dust, drinking untreated water, outdoor exposure to rain, and smoking [8]. Village tap water in Thailand is not chlorinated, and is commonly contaminated with *B*. *pseudomallei* [9]. Evidence-based guidelines for the prevention of melioidosis in Thailand recommend that residents and visitors to melioidosis-endemic areas avoid direct contact with soil and water, avoid outdoor exposure to heavy rain or dust clouds, wear protective gear such as boots and gloves when in direct contact with soil and environmental water, do not consume untreated water, and wash food to be eaten raw using boiled or bottled water [8]. These recommendations follow the ‘One Health concept’ (interdisciplinary collaborations and communications in all aspects of health care for humans, animals and the environment) and the national strategic plan currently being delivered by the Ministry of Public Health Thailand (MoPH) [10–12]. However, many people in Thailand currently do not follow the recommendations. For example, many still drink untreated water, and work in rice fields without protective gear [8]. Some patients also applied herbal remedies or an organic substance, such as soil or leaves, to open wounds [8].

Disease awareness and knowledge are important if prevention of melioidosis is to be successful. Based on anecdotal experience, most patients presenting to hospitals in northeast Thailand with melioidosis have never heard of the disease and do not know how to prevent infection (Personal communication, SD, ML, MM and DL). We hypothesized that public awareness of melioidosis is very low in Thailand. In March 2012, we organized the first meeting between melioidosis researchers and representatives from the MoPH to discuss the threat posed by melioidosis, the apparently low public awareness of melioidosis, and how to improve the implementation of preventive guidelines for melioidosis in Thailand [13]. This collaboration led to the first public engagement campaign, and a video clip contest entitled “Melioidosis, an infectious disease that Thais must know”, which was run between May and October 2012 [14]. In this study, we evaluated public awareness of melioidosis prior to a public engagement campaign, and gathered viewpoints of diabetics to the campaign’s video clips, to inform the development of educational tools about melioidosis in Thailand.

## Methods

### Population-based questionnaire

A two-page questionnaire was developed to evaluate public awareness of melioidosis, and knowledge about its prevention in the general population in Thailand (Appendix 1). Respondents were asked to provide information on gender, age, and highest level of education. To reduce response bias, we embedded questions about awareness of melioidosis amongst a group of common infectious diseases in the following order: HIV/AIDS, tuberculosis, melioidosis, malaria, leptospirosis, dengue, influenza, and bird flu. The level of public awareness regarding the list of common infectious diseases were categorized as follows: (1) never heard of the disease, (2) have heard of the disease but had no further knowledge, and (3) know about the disease. Participants were provided with a list of risk factors and asked whether they thought that each of these increased the risk of acquiring infectious diseases. To reduce the response bias, the word “melioidosis” was not written in this section, and questions were related to the wider list of infectious diseases. For example, we asked the participant, “Do you think that exposure to soil, for example by farming, gardening and walking in the mud, increases the risk of getting infectious diseases?” The level of belief regarding the risk of each factor were categorized as follows: (1) highly increases risk, (2) increases risk, (3) does not increase risk, and (4) do not know. The answer “highly increases risk” and “increases risk” were grouped as “yes”, and the answer “does not increase risk” and “do not know” were grouped as “no” in the analysis. Participants were then provided with a list of preventive behaviours and asked whether they thought that each of these could prevent them from acquiring infectious diseases. For example, we asked the participant, “Do you think that wearing protective gear such as rubber boots and rubber gloves during exposure to soil could protect you from getting infectious diseases?” The level of belief regarding the effectiveness of each preventive behaviour were categorized as follows: (1) highly effective, (2) effective, (3) not effective, and (4) do not know. The answer “highly effective” and “effective” were grouped as “yes”, and the answer “not effective” and “do not know” were grouped as “no” in the analysis. To evaluate the level of inaccurate knowledge about the direct application of herbal remedies or an organic substance to open wounds, these were added into the list of preventive behaviours in the questionnaire. The questionnaire was developed by PC and DL.

The study sample was drawn from the adult population (age >15 years old) from across Thailand. As of March 2012, Thailand consisted of 77 provinces (including Bangkok), 928 districts (range from 3 to 50 districts per province) and had a population of 64 million. The study team used the service provided by Suan Dusit Poll (http://dusitpoll.dusit.ac.th, Suan Dusit University, Thailand), who delivered our study questionnaire in a face-to-face interview to five randomly selected adults on the main street in each district (targeted sample size 4,640). Questionnaires were administered between 1 March and 31 April 2012. Questionnaires could not be delivered in 3 provinces in the South (including Yala, Pattani and Narathiwas) due to political and security problems during the study period. The questionnaire including a question on the highest education level of the respondent, categorized into no schooling completed, elementary education (aged 6 to 11), secondary education (aged 12 to 17), and undergraduate degree (usually aged 18 to 21). All data files are available in appendix 2.

### Development of the video clips

The video clips were selected as a potential education tool to increase awareness of melioidosis during the first meeting between melioidosis researchers and representatives from the MoPH. A national video clip competition entitled “Melioidosis, an infectious disease that Thais must know” was launched to generate video clips and ran between May and October 2012 [13]. Details of the competition are available at http://www.melioidosis.info/melioidclip.aspx [in English] and http://www.facebook.com/melioid [in Thai]. In brief, the public was invited to submit a short video clip about melioidosis via YouTube. The contest was announced via Facebook, direct contact to all schools and universities in Thailand, and by a press release by the Faculty of Tropical Medicine, Mahidol University, Thailand that was broadcast on multiple television channels, newspapers and magazines in Thailand. Information about melioidosis was provided on the website http://www.melioidosis.info/th. Facebook was selected as one of the communication channels due to its popularity in Thailand [15]. Forty-five video clips were submitted, which were judged in the first round by the contest organizing committee including PC, NW, PW, RK and DL. The best 12 video clips selected in the first round were used for focus group interviews.

### Focus Group interviews

Focus group interviews were conducted to evaluate perceptions of the video clips to inform the development of an educational tool for the diabetic population in northeast Thailand. Eligible participants were adults aged 18–60 years old with a diagnosis of diabetes mellitus for at least three months. Diabetics in northeast Thailand were selected as the study population because they are at the highest risk of melioidosis and are the target population for prevention [7]. Diabetics with a history of melioidosis were excluded. Participants were randomly selected from those who came to the diabetic clinics on the day. The focus group interviews were stopped after there were no new viewpoints in the discussion, indicating that the focus group interviews reached a saturation point. It was assumed that a focus group interview would enrich the data, as the participants would be able to compare and discuss their viewpoints within the group. Focus group interviews were conducted during November 2012 at Warinchamrab hospital, Warinchamrab district, Ubon Ratchathani province, northeast Thailand where melioidosis is highly endemic.

A total of 10 focus groups were held, each containing between 5–8 diabetics and lasting about 90 minutes. Six video clips were presented at the beginning of each session. After watching the video clips, each participant was asked to give a score to each clip before the focus group discussion. Focus group discussions were recorded in video and audio formats, and detailed notes were taken by NW. PC acted as a moderator and asked participants for further explanation as necessary. Audiotapes and videotapes of each focus group were reviewed by PC, NW and DL. Supplementary notes and quotations were added to the original field notes to ensure that all relevant participant comments and ideas were captured. Field notes and quotations were coded using an inductive analysis approach to identify categories of responses. The funders had no role in study design, data collection and analysis, decision to publish, or preparation of the manuscript.

### Ethics

Approval for the study was obtained from the Oxford Tropical Research Ethics Committee (OXTREC), University of Oxford, United Kingdom, and the Ethics Committee of the Faculty of Tropical Medicine, Mahidol University, Bangkok, Thailand. For the population-based questionnaire, verbal consent was obtained from each participant prior to the interview. For the focus group interview, written informed consent was obtained from each participant prior to each focus group session.

### Statistical Analysis

Chi-square test was used to compare categorical variables. All analyses were performed using the STATA version 12.0 (StataCorp LP, College station, Texas).

## Results

### Public awareness of melioidosis in Thailand

A total of 4,203 adults completed the questionnaire. The sample consisted of 954 (22.7%) men and 3,249 (77.3%) women with a median age of 36 years (interquartile range, 28 to 47; range, 13 to 80 years). There were 867 (20.6%), 1,566 (37.3%), 1,162 (27.6%) and 608 (14.5%) participants from north, northeast, central and south of Thailand, respectively. Level of education (categorized as no schooling completed, elementary education, secondary education, or undergraduate degree) was reported by 426 (10.6%), 611 (15.2%), 939 (23.3%) and 2,058 (51.0%) participants, respectively, while 169 (4.0%) did not answer the question about education. Age and geographical regions of the study participants were comparable to the general population in Thailand (Table 1) [16]. However, we noted that study participants were more likely to be female, and had higher education than the general population in Thailand (both p<0.001).

**Table 1:** Characteristics of 4,203 adult participants who completed the questionnaire about awareness and knowledge of common infectious diseases in Thailand.

**Characteristics**	**Study participants (N = 4,203)**	**Adult population in Thailand (N = 55,947,500)** *
**Male gender**	954 (22.7%)	49.0%
**Median age in year (IQR, range)**	36 (28–47, 13–80)	Age group 35–39 years old
**Geographical regions**		
North	867 (20.6%)	17.6%
Northeast	1,606 (38.2%)	28.8%
Central	1,162 (27.6%)	27.6%
South	568 (13.5%)	13.4%
**Highest education level** **		
No schooling completed	426 / 4,034 (10.6%)	28.1%
Grade 6	611 / 4,034 (15.2%)	19.3%
Grade 12	939 / 4,034 (23.3%)	33.1%
Undergraduate degree or higher	2,058 / 4,034 (51.0%)	10.1%

* Characteristics of participants were compared with the national data in 2010 reported by the National Statistical Office Thailand [16].

** 169 participants did not answer the question about education.

**Fig 1:**
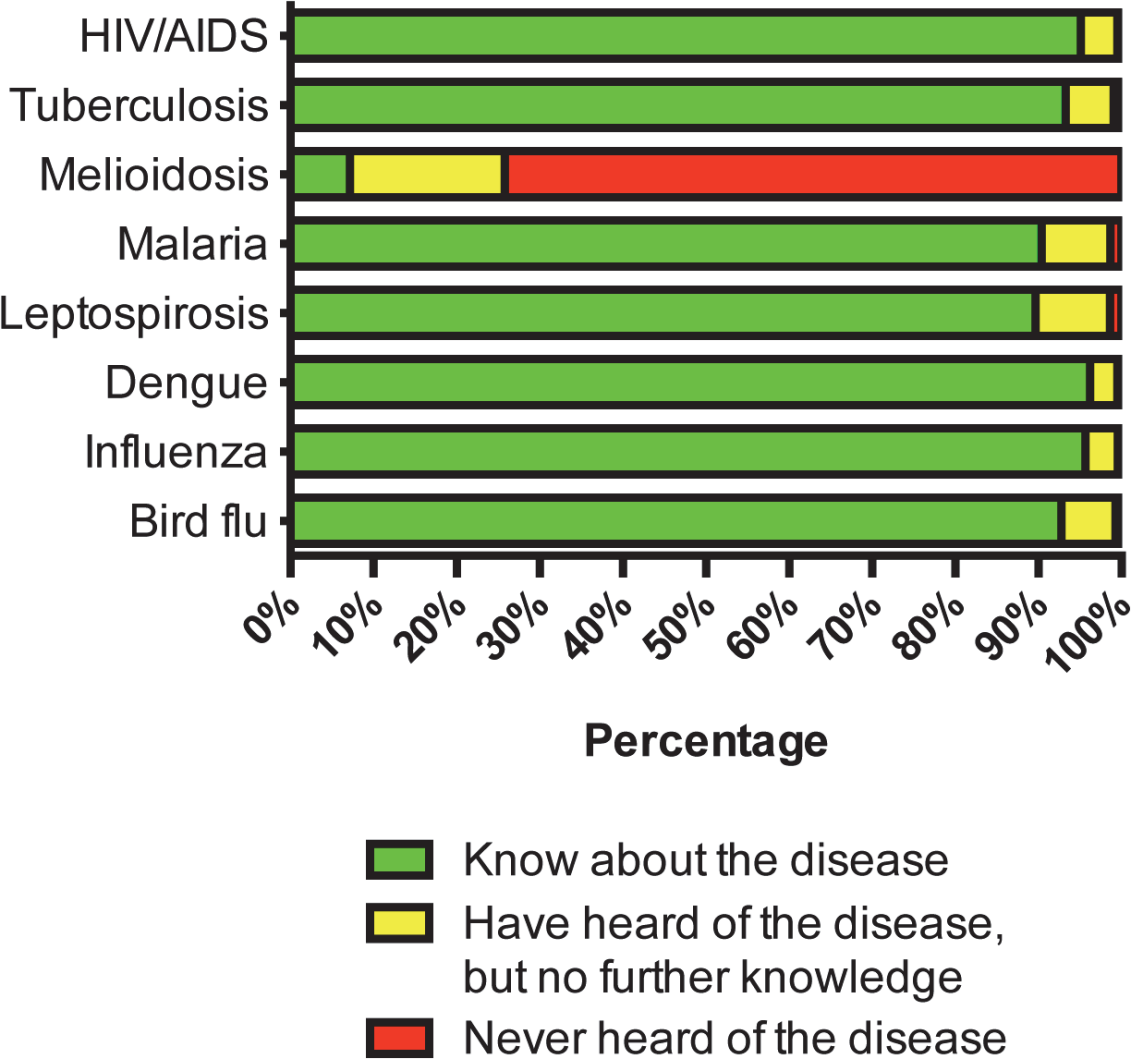
Awareness of common infectious diseases in Thailand (n = 4,203).

Nearly all participants (>99%) reported that they had heard or knew of common infectious diseases, including HIV/AIDS (99.5%), tuberculosis (99.1%), malaria (98.6%), leptospirosis (99.5%), dengue (99.5%), influenza (99.6%), and bird flu (99.3%). However, for melioidosis, 3,177 (74%) participants reported that they had never heard of the disease, with 783 (19%) responding that they had heard of this but had no further knowledge about melioidosis, and only 303 (7%) responding that they knew about melioidosis (Fig. 1). A higher proportion of participants from the northeastern regions where melioidosis is endemic had heard or knew of the disease compared with participants from other regions (28.4% v.s. 24.3%, p = 0.003). A higher level of education was associated with having heard or known of melioidosis (p<0.001, Table 2). For example, 30.4% of participants with an undergraduate degree had heard or knew of melioidosis compared to only 9.9% of participants with no schooling completed.

**Table 2:** Awareness of melioidosis in Thailand.

**Characteristics**	**N**	**Have never heard of melioidosis**	**Have heard or knew of melioidosis**	**P value**
**Gender**				
Male	954	709 (74.3%)	245 (25.7%)	0.90
Female	3,249	2,408 (74.1%)	841 (25.9%)	
**Age**				
Younger than 40 years old	2,464	1,804 (73.2%)	660 (26.8%)	0.10
Older than 40 years old	1,739	1,313 (75.5%)	426 (24.5%)	
**Geographical regions**				
North	867	654 (75.4%)	213 (24.6%)	0.03
Northeast	1,606	1,150 (71.6%)	456 (28.4%)	
Central	1,162	879 (75.7%)	283 (24.4%)	
South	568	434 (76.4%)	134 (23.6%)	
**Highest education level** *				
No schooling completed	424	384 (90.1%)	42 (9.9%)	<0.001
Grade 6	611	501 (82.0%)	110 (18.0%)	
Grade 12	939	692 (73.7%)	247 (26.3%)	
Undergraduate degree or higher	2,058	1,432 (69.7%)	626 (30.4%)	

* 169 participants did not answer the question about education.

**Fig 2:**
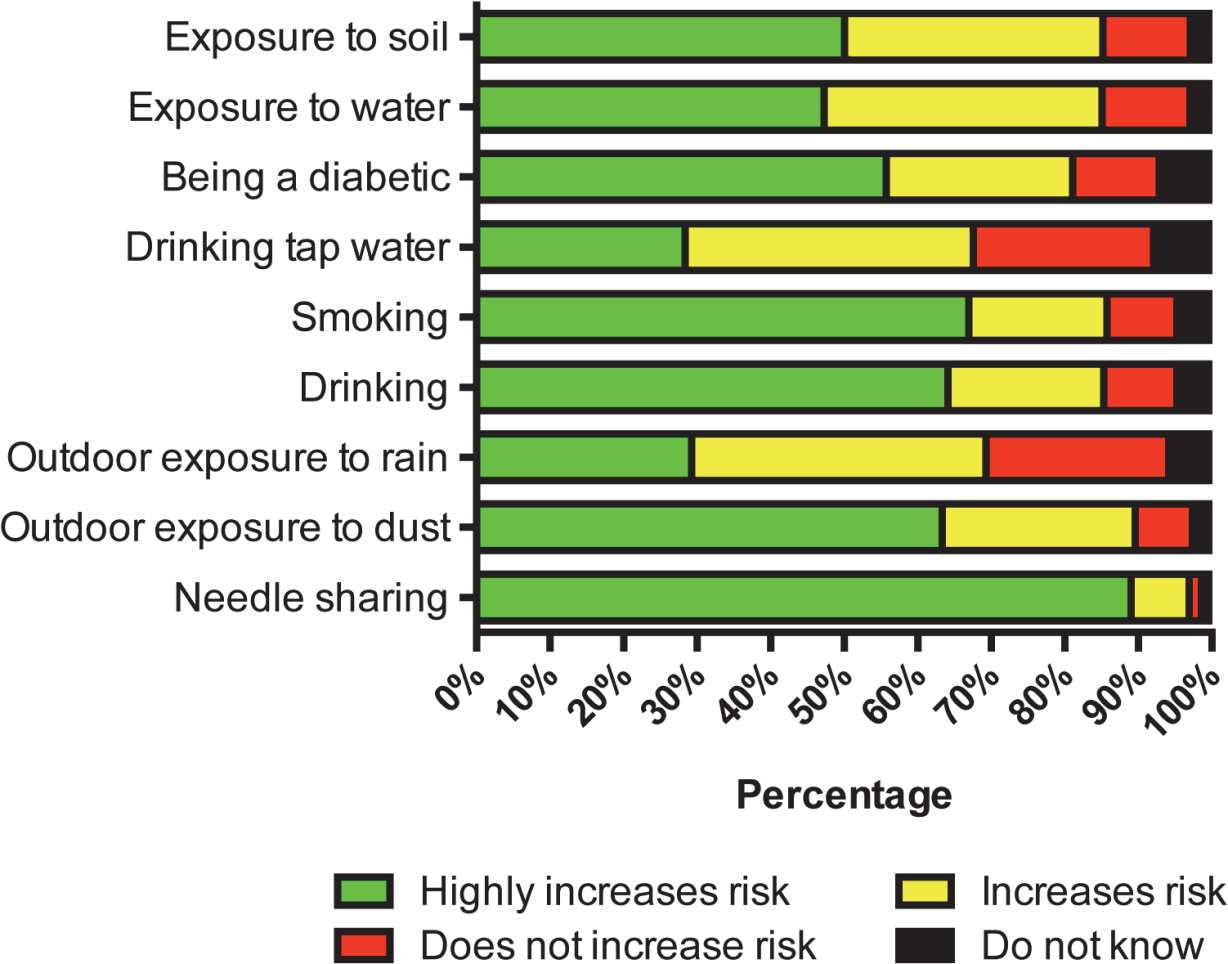
Knowledge about risk factors for common infectious diseases in Thailand (n = 4,203).

For risk factors of acquiring infectious diseases in general, most participants (>80%) thought that the risk increased with exposure to soil (85.2%), exposure to water (85.1%), being diabetic (81.1%), smoking (85.7%), drinking (85.3%), outdoor exposure to dust (89.6%), and needle sharing (96.9%). However, less than 80% of the participants thought that the risk of acquiring infectious diseases increased by drinking tap water (67.6%), or having outdoor exposure to rain (68.3%) (Fig. 2).

**Fig 3:**
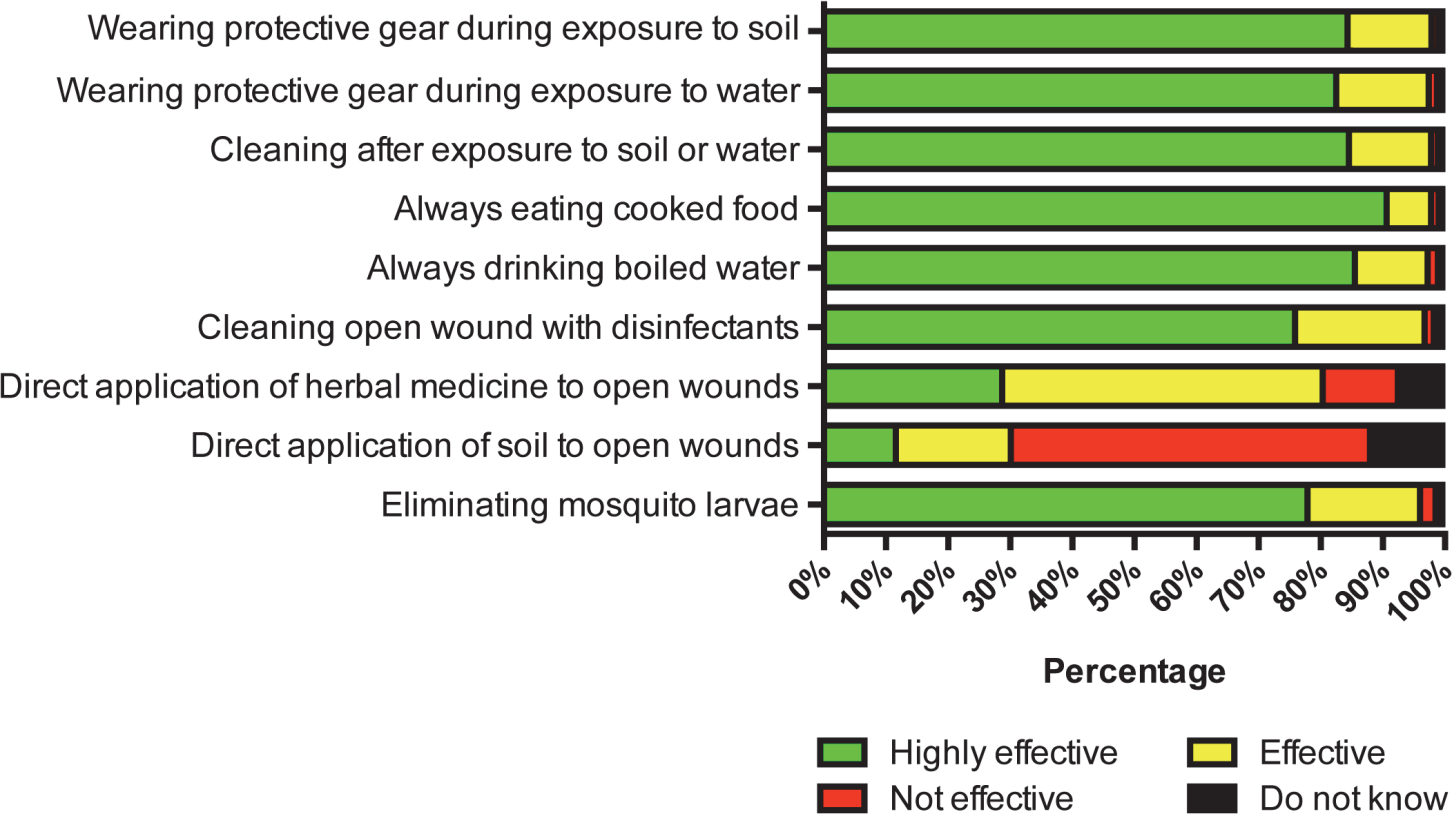
Knowledge about preventive measures for common infectious diseases in Thailand (n = 4,203).

For preventive measures, more than 95% of participants thought that infectious diseases in general could be prevented by wearing protective gear during exposure to soil (97.9%) or environmental water (97.4%), by cleaning after exposure to soil or water (97.8%), eating cooked food (97.8%), drinking boiled water (96.8%), cleaning open wounds with disinfectants (96.8%), and eliminating mosquito larvae (96.0%). However, many participants wrongly thought that applying herbal remedies (80.4%) or soil (30.1%) directly to open wounds could prevent infectious diseases (Fig. 3).

### Focus Group discussion about video clips of melioidosis

A total of 71 diabetic patients without a history of melioidosis participated in the ten focus groups. Overall, 21 (30%) were male and 50 (70%) were female. The median age of participants was 52 years (interquartile range, 48 to 55; range, 35 to 60 years). Sixty-eight participants (96%) had never heard of melioidosis, and 3 (4%) had heard of the disease but had no further knowledge. The topics of the focus groups were defined a priori and included (1) general viewpoints about video clips, (2) potential use of video clips to raise public awareness of melioidosis, (3) potential use of video clips to provide education about melioidosis prevention, and (4) responses and obstacles to the recommended preventive guidelines.

#### General viewpoints about video clips

In general, participants felt that using video clips was a good idea and suggested that these should be shown to other diabetic patients in Thailand. All participants valued the information provided by the video clips.

“We should show these video clips to other patients waiting downstairs to see the doctor” (Female participant, session 1)

“This (the video clip) is good. It can be used to inform (my) friends and everyone in the country (about the disease).” (Male participant, session 3)

In most sessions, participants suggested that they could understand and relate to the video clips better when these were spoken in the local dialect, and in simple words without the use of medical terms.

“If you want Isan (northeast) people to understand, a local dialect (Isan) should be used so that we can understand.” (Male participant, session 6)

“(The video clip) should use simple words for Isan people. It should not use medical terms as they are not understandable.” (Male participant, session 2)

Most participants felt that the video clips should not be narrated in a lighthearted fashion, such as in a cartoon clip or through an entertaining story. However, some participants liked the cartoon clip and found that it was easy to understand.

“This disease is dangerous but they (the presenters) are making it (the video clip) look entertaining. So, it might make the audience think that it (the disease) is not as dangerous as it is.” (Male participant, session 3)

“Isan (northeast) people may not understand cartoons, think that it (the disease) is a funny matter, and may not pay attention to it.” (Male participant, session 6)

“The cartoon clip is attractive. I understand (the disease)” (Male participant, session 2)

The other common general viewpoint was that doctors should deliver the key messages as rural people put their trust in them.

“Doctors must be the ones who speak. Most local people believe in doctors” (Male participant, session 6)

#### Potential use of video clips to raise public awareness

Most participants had never heard of the disease name, and found that the video clips were very useful in informing them about this.

“Is it rheumatoid disease? (I have) heard of only rheumatoid.” (Female participant, session 1)

“The doctor has not said anything about this disease yet. I have never heard of it” (Female participant, session 3)

“This is a scary disease. I just know of it now” (Female participant, session 10)

#### Potential use of video clips to provide education about melioidosis prevention

Participants stated that some video clips provided good education about the prevention of melioidosis.

“This video clip makes us picture clearly why we should wear (rubber boots)” (Male participant, session 7)

“I like the video clips. They are informative. They show examples (of recommended preventive behaviours).” (Female participant, session 4).

Participants also stated that they would follow recommendations for prevention after watching the video clips, including the difficult or time-consuming recommendations such as wearing boots while working in the rice fields and boiling water before drinking.

“It (the video clip) provides recommendations. We will try to follow what they recommended” (Female participant, session 9)

“(After watching the video clips), I will try to wear rubber boots” (Male participant, session 1)

“If it (wearing boots) is needed, I will do it” (Female participant, session 4)

“This video clip is good about boiling water. It makes people know that water coming out of the tap is not clean. Now I will boil the water as I know that it (boiled water) will not contain germs” (Male participant, session 7)

#### Obstacles to the recommended preventive guidelines

Most participants thought that the biggest obstacle to following preventive guidelines was to wear protective gear during exposure to water or soil, since wearing rubber boots made it difficult to walk in muddy paddy fields, and wearing rubber gloves made it difficult to plant rice. The problem of becoming hot while wearing rubber gear was also raised in most sessions.

“It is impossible for farmers to wear boots while working in the rice paddies. The mud sucks the boots. If it is just for walking on the road, we can wear boots. However, when working in rice paddies, only walking bare foot is possible. We cannot work while wearing boots.” (Male participant, session 4)

“Boots might be possible, but rubber gloves are impossible” (Female participant, session 1)

“It is impossible to wear rubber gloves. It’s hot.” (Female participant, session 9)

We found that many participants drank water without treatment, even though they knew that drinking boiled water was better, explaining that they do not have time to boil water.

“I know (that I should drink boiled water), but I do not do it.” (Female participant, session 9)

“I don’t boil (water). (I) work in the rice fields, (and I) can’t boil water in time. After working, I am tired. When I get back, I drink water immediately. (I) can’t boil water in time.” (Female participant, session 1)

## Discussion

Our study has shown that public awareness of melioidosis in Thailand is very low, indicating a need to increase knowledge about its prevention. Video clips were responded to positively, suggesting that they could serve as a useful medium to educate and promote prevention of melioidosis. The results of our focus group study suggested that video clips should be presented in the local dialect using simple words rather than medical terms, in a serious manner, with a doctor presenting the facts, and having detailed pictures of each recommended prevention method. These findings will contribute towards the future development of educational tools about melioidosis in Thailand.

The finding that 74% of adults in Thailand have not heard of melioidosis is not surprising. To our knowledge, information about melioidosis is rarely given to the public by the mass media, including television, newspapers and radio stations. Basic information about melioidosis and its prevention is also not taught in schools in Thailand. By contrast, public awareness of other common infections including HIV/AIDS, tuberculosis, malaria, leptospirosis, dengue and influenza, which are taught in schools and frequently mentioned by the national media, is very high. The result is consistent with our clinical observations that many melioidosis patients have not heard about the disease when they first present to hospital with this infection [8], and with our focus group findings that most participants have not heard of melioidosis.

As people are not aware of the disease, it is not surprising that they do not know about how to prevent the disease. Finding that many people think that drinking tap water does not increase the risk of acquiring infectious diseases need urgent consideration by policy makers. Our previous environmental study showed that 10% of village tap water samples were contaminated with *B*. *pseudomallei* [9], and that drinking untreated water was associated with developing melioidosis [8]. Misunderstanding that direct application of herbal remedies or an organic substance such as soil to open wounds can prevent infectious disease also needs to be addressed. Neither is sterile and could contain pathogenic organisms, including *B*. *pseudomallei*. Our previous study also showed that the application of herbal remedies or an organic substance to open wounds was associated with developing melioidosis [8]. Policy makers should aim to provide more education about prevention of infectious diseases, together with improving the quality of tap water provided to village people so that it does not contain any pathogenic organisms.

Changing behaviour requires a systematic approach. In circumstances where awareness and knowledge are low, increasing the levels of these is fundamental for people to change their routine patterns of behaviour [17]. Our study also found that some participants in the focus group interviews knew about preventive recommendations, but did not follow them. This is consistent with the proposed model by Michie et al. that changing behaviour may require changing not only capability (including awareness and knowledge), but also opportunity and motivation [18]. Another major concern raised by the focus group participants was that rubber boots are hot and make walking difficult in muddy rice fields, and that rubber gloves are also hot and difficult to use while planting rice. The problem of boots and gloves was also raised in a study on leptospirosis prevention in developing countries such as Sri Lanka [19]. Interventions should be designed based on a careful analysis of what is required to change behaviour in each specific target population in its particular context [20]. Further studies should also focus on developing and trialing specifically designed boots and gloves that could allow farmers to walk easily in muddy paddy fields and comfortably in hot weather.

The use of YouTube for sharing of educational videos via the Internet has been increasingly used and objectively evaluated [21–23]. It is likely that through increasing use of cheap smartphones the Internet will be easily accessible to the public, even in developing countries. The availability of educational videos on YouTube could be helpful to a range of groups, including people with melioidosis, the general population, hospital staff and policy makers. One of the awarded winning video clips from our contest had 30,000 views by December 2012 when the award was given to the developer, and had 34,000 views by June 2014 (http://youtu.be/l1VxcZTw-BU). This suggests that there are still people watching the video clip after the competition, and highlights the benefit of the YouTube as a platform for providing educational videos to the general public.

A limitation of this study is that questionnaire participants had relatively high educational levels compared with the general population in Thailand. The questionnaires were distributed by Suan Dusit Poll, and study samples may have over-represented people living in the center of each district, and under-represented people living in more remote, rural areas. It is possible that male gender, and people with a low level of education, were more likely to decline the invitation to complete the questionnaire. Nonetheless, this sampling bias suggests that awareness and knowledge might be even lower in the general population than our results suggest. Using competitions and targeting schools and universities to generate video clips, we found that many used medical jargon in video clips without considering their target population who spoke local dialects and preferred simple language. However, a few video clips fulfilled these criteria and could be used to educate target populations. These could be made to video compact disk (VCD) and distributed to every primary care unit (PCU) in Thailand, which has ready access to televisions and CD players that can be used to show video clips to the target population. Other educational tools for rural populations such as pamphlets and group education should also be considered for future interventions.

Our findings suggest that policy makers together with program planners in education, health and workplaces should urgently consider raising public awareness and knowledge of melioidosis and its prevention, and systematically develop interventions to increase preventive behaviours based on careful, theoretically-informed analyses of the target behaviour in the context of the target populations.

## Supporting Information

S1 Text Questionnaire. (DOCX)

S1 Table Data file. (XLS)
