# Supplementary material for: Public Awareness of Melioidosis in Thailand and Potential Use of Video Clips as Educational Tools
Source: PLoS One. 2015 Mar 24;10(3):e0121311. doi: 10.1371/journal.pone.0121311 (PMC4372587; doi:10.1371/journal.pone.0121311)
Supplement: S1 Text — (DOCX) [file pone.0121311.s001.docx]

**Text S1: QUESTIONNAIRE (English translation from Thai original)**

**Questionnaire Completion Guideline**

We would like to ask for 5 minutes of your time to complete this questionnaire. Please answer the questionnaire as truthfully as you can. Thank you very much for your assistance and the knowledge gained from this questionnaire will be used to develop educational learning tools on infectious disease for the general public.

***General Information***

1. Gender Male Female
2. Age _______ years
3. Highest level of education

No schooling completed Grade 6 or equivalent Grade 9 or equivalent

Grade 12 or equivalent Bachelor degree or equivalent Higher than Bachelor

1. Address District______________ City______________

***Have you ever heard of these infectious diseases?***

| 1. AIDS | Never heard of the disease | Have heard of the disease but do not know about it | Know of the disease |
| --- | --- | --- | --- |
| 1. Tuberculosis | Never heard of the disease | Have heard of the disease but do not know about it | Know of the disease |
| 1. Melioidosis | Never heard of the disease | Have heard of the disease but do not know about it | Know of the disease |
| 1. Malaria | Never heard of the disease | Have heard of the disease but do not know about it | Know of the disease |
| 1. Leptospirosis | Never heard of the disease | Have heard of the disease but do not know about it | Know of the disease |
| 1. Dengue fever | Never heard of the disease | Have heard of the disease but do not know about it | Know of the disease |
| 1. Influenza | Never heard of the disease | Have heard of the disease but do not know about it | Know of the disease |
| 1. Bird flu | Never heard of the disease | Have heard of the disease but do not know about it | Know of the disease |

***Do you think that these behaviors increase the risk of getting infectious diseases?***

| 1. Exposure to soil, for example by farming, gardening and walking in the mud | Highly increases risk | Increases risk | Does not increase risk | Do not know |
| --- | --- | --- | --- | --- |
| 1. Exposure to water, for example, fishing | Highly increases risk | Increases risk | Does not increase risk | Do not know |
| 1. Being a diabetic | Highly increases risk | Increases risk | Does not increase risk | Do not know |
| 1. Drinking tap water | Highly increases risk | Increases risk | Does not increase risk | Do not know |
| 1. Smoking | Highly increases risk | Increases risk | Does not increase risk | Do not know |
| 1. Drinking | Highly increases risk | Increases risk | Does not increase risk | Do not know |
| 1. Outdoor exposure to rain | Highly increases risk | Increases risk | Does not increase risk | Do not know |
| 1. Outdoor exposure to dust | Highly increases risk | Increases risk | Does not increase risk | Do not know |
| 1. Needle sharing | Highly increases risk | Increases risk | Does not increase risk | Do not know |

***Do you think that the following measure could protect you from getting infectious diseases?***

| 1. Wearing protective gear as rubber boots and rubber gloves during exposure to soil | Highly effective | Effective | Not effective | Do not know |
| --- | --- | --- | --- | --- |
| 1. Wearing protective gear such as rubber boots and rubber gloves during exposure to water | Highly effective | Effective | Not effective | Do not know |
| 1. Cleaning after exposure to soil or water | Highly effective | Effective | Not effective | Do not know |
| 1. Always eating cooked food | Highly effective | Effective | Not effective | Do not know |
| 1. Always drinking boiled water | Highly effective | Effective | Not effective | Do not know |
| 1. Cleaning open wounds with disinfectants | Highly effective | Effective | Not effective | Do not know |
| 1. Direct application of herbal medicine to open wounds | Highly effective | Effective | Not effective | Do not know |
| 1. Direct application of soil to open wounds | Highly effective | Effective | Not effective | Do not know |
| 1. Eliminating mosquito larvae | Highly effective | Effective | Not effective | Do not know |
